# Diverse Associations of Plasma Selenium Concentrations and SELENOP Gene Polymorphism with Metabolic Syndrome and Its Components

**DOI:** 10.1155/2020/5343014

**Published:** 2020-04-22

**Authors:** Li Zhou, Cheng Luo, Jiawei Yin, Yalun Zhu, Peiyun Li, Sijing Chen, Taoping Sun, Manling Xie, Zhilei Shan, Benfeng Cao, Xueting Hu, Ying Rong, Wei Yang, Xiaoqin Li, Aijun Tan, Liegang Liu

**Affiliations:** ^1^Department of Nutrition and Food Hygiene, Hubei Key Laboratory of Food Nutrition and Safety, School of Public Health, Tongji Medical College, Huazhong University of Science and Technology, Wuhan, China; ^2^Ministry of Education Key Laboratory of Environment and Health, School of Public Health, Tongji Medical College, Huazhong University of Science and Technology, Wuhan, China; ^3^Department of Neurology, Mayo Clinic, Rochester, MN, USA; ^4^Department of Nutrition, Harvard T.H. Chan School of Public Health, Boston, MA, USA; ^5^Department of Nutrition, Shandong Provincial Hospital Affiliated to Shandong University, Jinan, China; ^6^Zhuhai Center for Disease Control and Prevention, Zhuhai, China

## Abstract

The relationship between selenium and metabolic syndrome (MetS) has been discussed controversially, and limited studies have examined the associations of single nucleotide polymorphisms in selenoproteins genes with MetS. Hence, to examine the associations of plasma selenium concentrations and selenoprotein P rs7579 polymorphism with MetS, a case-control study of 1279 MetS cases and 1279 sex- and age- (±2 years) matched controls was conducted based on the baseline data of the Tongji-Ezhou Cohort study. Plasma selenium concentrations were measured by inductively coupled plasma mass spectrometry. MetS was defined using the definition of the Joint Interim Statement, adjusted for the Chinese population. In addition, the rs7579 polymorphism was genotyped by the Agena MassARRAY System. Plasma selenium concentrations in the MetS group were higher than in the control group (93.88 *μ*g/L (83.17-107.41) vs. 92.66 *μ*g/L (82.36-103.53), *P* < 0.05). Compared with quartile 4 (≥103.53 *μ*g/L), the multivariate-adjusted odds ratios (ORs) and 95% confidence intervals (CIs) associated with MetS were 0.79 (0.59-1.06) for quartile 1 (<82.36 *μ*g/L), 0.75 (0.56-1.01) for quartile 2 (82.37-92.66 *μ*g/L), and 0.61 (0.45-0.83) for quartile 3 (92.67-103.52 *μ*g/L). The cubic spline analyses revealed a U-shaped association between plasma selenium and MetS, with the lowest risk at around 93.69 *μ*g/L. Moreover, in cubic spline analyses, plasma selenium showed U-shaped associations with central obesity and high blood pressure, positive associations with hypertriglyceridemia and hyperglycemia, and a negative association with low high-density lipoprotein cholesterol. Additionally, both the GA and GA+AA genotype carriers were associated with increased ORs of MetS comparing with the GG genotype carriers. Our findings suggested a U-shaped association between plasma selenium and MetS and diverse associations between plasma selenium and components of MetS. Furthermore, our study found that the A allele of rs7579 was associated with higher odds of MetS. Further studies are needed to confirm our findings and elucidate the underlying mechanisms.

## 1. Introduction

One-third of Chinese adults have metabolic syndrome (MetS) [1], which is characterized by a cluster of metabolic abnormalities including abdominal obesity, hyperglycemia, hypertension, and dyslipidemia [2]. The combination of these closely related metabolic disorders increases the risk of cardiovascular diseases and type 2 diabetes [3]. Therefore, the prevention of MetS is clearly imperative, and identifying risk factors and understanding their mechanisms should be of high priority. Although the pathogenesis of MetS is not fully understood, several studies suggest that dietary and genetic factors may play roles in the development of MetS [4].

Selenium, an essential trace element acquired mainly from diet, is involved in several essential functions such as redox homeostasis and endocrine and metabolic activities [5]. As an essential element, low selenium is associated with several adverse health outcomes including cardiovascular diseases, cancer, and death [6]. Despite being essential, selenium is an oxidant at high concentration and its redox properties contribute to the production of excessive damaging reactive oxygen species (ROS), which induces oxidative stress [7]. Via this mechanism, high selenium also was associated with cardiometabolic diseases [8]. Therefore, a U-shaped association of selenium with cardiometabolic diseases has been suggested. However, previous data regarding selenium and MetS are conflicting: some studies find circulating selenium to be positively associated with MetS [9, 10], whereas others report nonsignificant association between selenium and MetS [11, 12], occasionally linking higher selenium with lower risk of MetS [13]. Additionally, data related to the dose-response association between selenium and MetS are limited.

In human beings, selenium achieved nutritional functions mainly through selenoproteins. Selenoprotein P (SELENOP), as the key selenium transporter in the body, has been linked to several metabolic processes [5]. In an experimental study, the administration of purified SELENOP impaired insulin signaling and dysregulated glucose metabolism [14]. Conversely, the downregulation of SELENOP expression improved systemic insulin sensitivity and glucose tolerance [14]. A previous study identified a single nucleotide polymorphism (SNP) located in the 3′-untranslated region of the SELENOP gene, namely rs7579, which influenced both SELENOP mRNA expression and protein level [15]. Further studies provided evidence to correlate this SNP with metabolic factors; the SNP was associated with serum lipids and insulin sensitivity index [16, 17]. However, to our knowledge, no study has reported the association of the SELENOP rs7579 polymorphism with MetS.

Therefore, based on the baseline data of the Tongji-Ezhou Cohort study, a large matched case-control study was conducted to examine the associations of plasma selenium concentrations and the SELENOP rs7579 polymorphism with MetS.

## 2. Methods

### 2.1. Study Population and Design

The Tongji-Ezhou Cohort study was launched in 2013 to investigate the associations of lifestyle, dietary factors, and genetic markers with chronic diseases in Ezhou, China. Between 2013 and 2014, 5533 adult residents were recruited, with a response rate of 96.6%. Data allowing the determination of the status of MetS was available for 4137 of the participants, and the prevalence of MetS was 33.6%. Among them, after excluding individuals with any clinically significant disease (*n* = 97) or those with missing plasma selenium concentrations (*n* = 256), 1279 members classified as MetS were included in the present study. Control individuals were randomly selected among participants without MetS, and 1 control was individually matched to each MetS case according to sex and age (±2 years). Totally, 1279 MetS cases and 1279 controls were included in the study analyses. The flow chart of participant recruitment and case-control selection is shown in Figure S1.

The study was approved by the Ethics and Human Subject Committee of Tongji Medical College. Written informed consent was obtained from each participant enrolled.

### 2.2. Data Collection

Baseline data including sociodemographic information, lifestyle, and health status were collected via semistructured questionnaires during face-to-face interviews. Smoking status was assessed as current, former, and never; a similar method was also utilized to evaluate the alcohol drinking status. Physical activity was defined as regular exercise for at least 60 min per week for more than half a year. Education level was grouped into three categories: none or elementary school, middle school, and high school or college. Body weight, standing height, and waist circumference were measured in light indoor clothing and without shoes. Resting systolic and diastolic blood pressure measurements were obtained in a seated position after 5 minutes of seated rest. A standard mercury sphygmomanometer was used for obtaining measurements. Body mass index (BMI) was calculated as weight in kilograms divided by height in meters squared.

After overnight fasting, venous blood samples were collected in ethylene diamine tetraacetic acid anticoagulative tubes and centrifuged at 1620 × g at 4°C for 5 minutes. Then, the plasma was separated and stored at -80°C for subsequent analyses of selenium and other blood variables. Fasting plasma triglycerides, total cholesterol, high-density lipoprotein cholesterol (HDL-C), low-density lipoprotein cholesterol (LDL-C), and fasting plasma glucose were obtained with automated bioassays.

### 2.3. MetS Case Definition

Individuals were classified as having MetS according to the Joint Interim Statement [18]. MetS was defined as the presence of 3 or more of the following risk factors: (1) central obesity—waist circumference ≥ 90 cm in men or ≥80 cm in women (following Chinese-specific cutoffs for abdominal obesity defined by the International Diabetes Federation) [19]; (2) hypertriglyceridemia—triglycerides≥1.70 mmol/L; (3) low levels of HDL-C—HDL − C<1.03 mmol/L in men or <1.30 mmol/L in women; (4) high blood pressure—blood pressure ≥ 130/85 mmHg or use of antihypertensive medication; (5) and high fasting glucose—≥5.56 mmol/L or current use of antidiabetic medication or self-reported history of diabetes.

### 2.4. Measurement of Plasma Selenium Concentrations

Plasma selenium concentrations were detected in the Ministry of Education Key Laboratory of Environment and Health at Tongji Medical College of Huazhong University of Science and Technology, using inductively coupled plasma mass spectrometry (Agilent 7700 Series, Tokyo, Japan), as described in our previous study [20]. Samples from cases and controls were randomly assayed. Prior to analysis, all samples were thawed and mixed thoroughly by vortex. Digestive solution was composed of 2% (*w*/*w*) HNO_3_, 0.04% (*w*/*w*) Triton X-100, and 1% (*w*/*w*) butan-1-ol. Then, samples (40 *μ*L plasma) were diluted with ultrapure water and digestive solution at a ratio of 1 : 19 : 20. The detection limit of selenium was 0.024 *μ*g/L, and the concentration of the lowest standard solution (0.02 *μ*g/L) was considered as the limit of quantitation. A calibration curve that ranged from 0.02 *μ*g/L to 100 *μ*g/L was used to determine the concentration of plasma selenium. In addition, 1 mg/L internal standard composed of germanium in nitric acid was pumped into the instrument with the calibration standards or preprocessed samples at a rate of 0.10 m/s. This is done to correct for the loss of analyte during sample inlet. For quality assurance, the certified reference materials ClinChek No. 8883 and No. 8884 human plasma controls for trace elements were analyzed in every 20 samples. The concentrations of selenium were 83.6 ± 2.9 *μ*g/L (certified: 81.4 ± 16.3 *μ*g/L) and 123.6 ± 4.2 *μ*g/L (certified: 120.0 ± 24.0 *μ*g/L) for No. 8883 and No. 8884, respectively. The intra-assay and interassay coefficients of variation of plasma selenium were both <5%.

### 2.5. Genotyping

Based on identified SELENOP polymorphism oci with cardiometabolic outcomes, SNPs with minor allele frequencies of more than 0.05 were selected according to both HapMap HCB and CHB data in dbSNP. Finally, one informative SELENOP SNP (rs7579) was selected to test the associations of SELENOP with MetS and its components. The SELENOP rs7579 polymorphism was genotyped by the MassARRAY System (Agena Bioscience iPLEX Assay, San Diego, United States). Genomic DNA was isolated in 1195 individuals (551 MetS cases and 644 controls) from the peripheral blood sample by a kit (Tiangen Biotech, Beijing, China). The sample DNA was amplified by a multiplex polymerase chain reaction for a locus-specific single-base extension reaction. The alleles were discriminated by mass spectrometry (Agena Bioscience, San Diego, United States). The rs7579 SNP was in Hardy-Weinberg's equilibrium (*P* > 0.05) for both case and control participants (data not shown).

### 2.6. Statistical Analysis

General characteristics were summarized as means ± standard deviations for parametrically distributed variables, medians (interquartile ranges) for nonparametrically distributed variables, and percentage for categorical variables. Differences of characteristics between cases and controls were examined by Student's *t* test (normal distribution) or Mann-Whitney's *U* test (nonnormal distribution) for continuous variables and chi-square tests for categorical variables. Logistic regression models were used to compute the odds ratios (ORs) and 95% confidence intervals (CIs) of MetS and its components. Plasma selenium concentrations were considered as continuous variables and categorized into quartiles according to their distribution among the control group (quartile 1: <82.36 *μ*g/L; quartile 2: 82.37-92.66 *μ*g/L; quartile 3: 92.67-103.52 *μ*g/L; and quartile 4: ≥103.53 *μ*g/L). Linear trend *P* values were estimated by modeling the median value of each quartile as a continuous variable. Potential confounding variables including sex, age, BMI, smoking status, alcohol drinking status, physical activity, and education level were adjusted. To test the interactions between selenium and sex in relation to MetS and its components, we introduced multiplicative interaction terms of plasma selenium quartiles and sex as continuous variables and added these variables to binary logistic regression models, and confounding variables mentioned above were adjusted. Likelihood ratio test with one degree of freedom was used to assess the significance of the interaction between plasma selenium concentration and sex, with a comparison of the likelihood scores of the two models with and without the interaction term. Because of skewed distribution of plasma selenium concentrations, the normal distribution was approximated by logarithmic transformation. In addition, the overall associations of logarithmic transformed plasma selenium with MetS and its components were estimated using restricted cubic splines with 5 knots at the 10th, 25th, 50th, 75th, and 90th percentiles of its distribution via Stata.

A likelihood ratio test was used to analyze the distribution of genotype for deviation from Hardy-Weinberg's equilibrium. We used binary logistic regression analysis to estimate the ORs and 95% CIs of MetS and its components with the rs7579 polymorphism. Furthermore, the associations of the rs7579 polymorphism with MetS and its components stratified by sex and the associations of plasma selenium with MetS and its components stratified by rs7579 genotypes were examined with the same method described in the sex-stratified analyses. Statistical power for associations of rs7579 polymorphisms with MetS and its components was calculated using QUANTO 1.2.4 (https://preventivemedicine.usc.edu/download-quanto/). Assuming a minor allele frequency of 0.26 and disease prevalence of 33%, we had 92% power to detect genetic effects at an OR of 1.50 in our samples. All statistical tests were two sided, and significance was set at *P* < 0.05. All analyses were conducted with SPSS 23.0 (SPSS Inc., Chicago, IL) and Stata version 12 (StataCorp LP, College Station, TX).

## 3. Results

### 3.1. Characteristics of the Participants

Table 1 summarizes the demographic and clinical characteristics of 2558 participants (1279 cases of MetS and 1279 controls). No significant differences in sex, age, education level, and lifestyle factors were observed between groups. The mean age of the MetS cases and control individuals was 55.56 ± 10.46 and 55.36 ± 10.53 years, respectively, and 64.0% of the participants were men. Compared with control participants, individuals with MetS had higher waist circumference and blood pressure, higher levels of fasting plasma glucose, and more unfavorable lipid profiles (lower HDL-C and higher triglyceride levels). Higher plasma selenium concentrations were observed in MetS cases than in control participants (93.88 *μ*g/L (83.17-107.41) vs. 92.66 *μ*g/L (82.36-103.53), *P* < 0.05).

### 3.2. Associations of Plasma Selenium Concentration with MetS and Its Components

The associations of plasma selenium concentration with MetS and its components obtained from logistic regression analyses were displayed in Table 2. Compared with quartile 4 (≥103.53 *μ*g/L), after adjustment for sex, age, BMI, smoking, drinking, physical activity, and education level, the ORs (95% CIs) associated with MetS were 0.79 (0.59-1.06) for quartile 1 (<82.36 *μ*g/L), 0.75 (0.56-1.01) for quartile 2 (82.37-92.66 *μ*g/L), and 0.61 (0.45-0.83) for quartile 3 (92.67-103.52 *μ*g/L) (Table 2). In the logistic regression models, plasma selenium concentrations were positively associated with odds of hypertriglyceridemia and hyperglycemia, and inversely associated with odds of low HDL-C (*P* value for trend < 0.05). Additionally, we observed a nonmonotonic relationship between plasma selenium and central obesity, with a significantly decreased OR (OR: 0.67 (95% CI: 0.50-0.89)) of central obesity observed only in quartile 3 comparing with quartile 4. Nonetheless, the association between plasma selenium and high blood pressure was not statistically significant (*P* > 0.05). We further examined associations of plasma selenium with MetS and its components stratified by sex (Table S1). Notably, the inverse relationship between plasma selenium and low HDL-C seemed stronger in men than in women, and a significant interaction between plasma selenium and sex in relation to low HDL-C was found.

**Figure 1:**
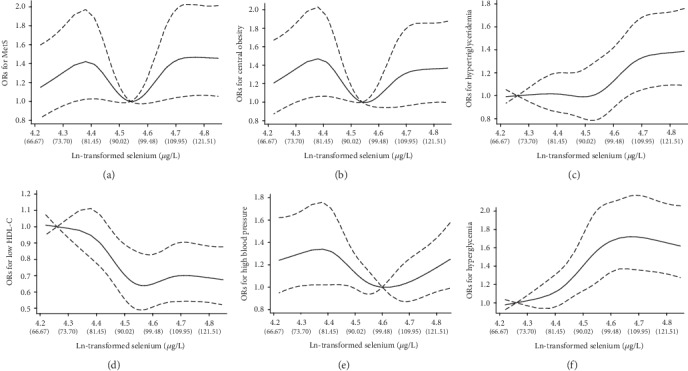
Representation of restricted cubic spline logistic regression models for ln-transformed selenium and risk of MetS and its components. The solid lines represent adjusted odds ratios of (a) MetS, (b) central obesity, (c) hypertriglyceridemia, (d) low HDL-C, (e) high blood pressure, and (f) hyperglycemia based on restricted cubic splines for ln-transformed selenium with 5 knots at the 10th, 25th, 50th, 75th, and 90th percentiles of its distribution. And dashed lines surrounding the solid lines represent the 95% confidence intervals. Confounding factors including sex, age (years), and body mass index (kg/m^2^), smoking (current, former, and never), drinking (current, former, and never), vigorous activity (yes or no), and education level (none or elementary school, middle school, and high school or college) were adjusted. OR: odds ratio; MetS: metabolic syndrome.

In cubic spline models, plasma selenium showed a U-shaped association with MetS (Figure 1(a)). The OR of MetS showed an increase trend before plasma selenium reached 79.38 *μ*g/L. Then, the OR of MetS decreased steeply and reached the lowest value when plasma selenium was about 93.69 *μ*g/L. After plasma selenium reached 93.69 *μ*g/L, the OR of MetS increased significantly, but levelled off at around 109.95 *μ*g/L. As for the association of selenium with central obesity and high blood pressure (Figures 1(b) and 1(e)), the cubic spline analyses presented similar U-shaped associations to that of the Se-MetS association. From the spline models, we could see positive associations of selenium with hypertriglyceridemia and hyperglycemia (Figures 1(c) and 1(f)). In addition, the spline results presented a negative association between selenium and low HDL-C (Figure 1(d)).

### 3.3. Associations of rs7579 Polymorphism with MetS and Its Components

The general characteristics of the 1194 genotyped individuals were presented in Table S2. Minor allele frequencies (A allele) of rs7579 in control and MetS groups were 25.3% and 26.9%, respectively. There was a significant association between rs7579 and MetS (Table 3). Compared with the GG genotype, after adjustment for sex, age, BMI, smoking, drinking, physical activity, and education level, the GA and GA+AA genotypes were associated with increased odds of MetS (ORs (95% CIs): 1.42 (1.06-1.91) and 1.35 (1.03-1.77), respectively). However, different from the variable associations between selenium and components of MetS, no significant association between rs7579 and any component of MetS was observed in the current study (*P* > 0.05).

The nonlinear association between plasma selenium and MetS was not modified by rs7579 genotypes (Table 4). Moreover, no significant interaction was found between selenium and rs7579 in relation to any component of MetS except for hyperglycemia. The positive association between plasma selenium and hyperglycemia seemed to be stronger in AA genotype carriers than in GG and GA genotype carriers. We also performed stratified analyses by sex (Table S3). The associations between the rs7579 polymorphism and MetS, as well as components of MetS, were not significantly different between women and men. In addition, the interactions between the rs7579 polymorphism and sex in relation to MetS and its components were not significant (*P* > 0.05).

## 4. Discussion

In this matched case-control study among a Chinese population, plasma selenium showed a U-shaped association with MetS. The risk was lowest when plasma selenium was about 93.69 *μ*g/L, higher and lower from this showed increased risk. We also observed U-shaped associations of plasma selenium with central obesity and high blood pressure. In addition, higher plasma selenium was positively associated with odds of hypertriglyceridemia and hyperglycemia, and negatively associated with odds of low HDL-C. As for the SELENOP rs7579 polymorphism, our study firstly revealed the risk allele A with MetS. These trends persisted after adjusting for confounding variables.

Our unique finding adds to the body of literature exploring the selenium-MetS association. Some studies reported positive association between selenium and MetS risk. Recently, a case-control study from China found a positive relation between selenium and MetS [21]. In that study, the median plasma selenium concentration in the MetS group was 146.3 *μ*g/L, which was much higher than that in our study (93.88 *μ*g/L). In our study, plasma selenium above 93.69 *μ*g/L was positively associated with MetS, which was in line with that study. Findings from the Third National Health and Nutrition Examination Survey demonstrated that MetS status was not significantly associated with serum selenium [11]. However, that study assumed a linear relation between selenium and MetS, which precluded it to explore the nonlinear selenium-MetS association. Moreover, some studies revealed negative association between selenium and MetS. For example, a case-control study in Iran adolescents found an inverse relationship [13]. In that study, the median of plasma selenium was 84.32 *μ*g/L for the controls and 81.92 *μ*g/L for the cases, and each SD increment of serum selenium was associated with 2% (95% CI: 0-3%) lower odds of MetS. In our study, higher plasma selenium was associated with lower odds of MetS when plasma selenium was below 93.69 *μ*g/L, which was consistent with that study. Therefore, the discrepancies in results might be partly related to the different distributions of circulating selenium and the nonlinearity of the selenium-MetS relationship.

Circulating selenium is a reliable biomarker of selenium intake [22]. Contrary to many other elements, the intake of selenium varies enormously worldwide, ranging from deficient to toxic concentrations that lead to adverse health outcomes [23]. Intakes are high in the USA, Japan, and Venezuela, and lower in Europe [23]. China has both selenium-deficient and selenium-excessive areas [23]. A previous review revealed that the means of blood selenium range from 27 to 3200 *μ*g/L in China [24]. Our study was conducted in a selenium appropriate area in China, and medians of plasma selenium concentration were 92.66 *μ*g/L (82.36-103.53) for controls and 93.88 *μ*g/L (83.17-107.41) for MetS cases in our study, which were different from those conducted in the high selenium areas or low selenium areas of China. For example, a study conducted in Enshi, which is well known for selenium poisoning in China, reported a blood selenium concentration of 3248 *μ*g/L [25]. Considering the nonlinearity of the selenium-MetS relationship and the various selenium status, our findings might not be generalized to people with different selenium status. And large prospective studies among subjects with different selenium status are warranted to further investigate the selenium-MetS association.

Besides the selenium-MetS association, our study also found variable associations between selenium and components of MetS. The positive association between selenium and hyperglycemia supports previous evidence suggesting that high selenium may have a diabetogenic effect. A recent meta-analysis revealed a positive association between selenium exposure and diabetes both in epidemiologic and experimental studies [26]. The association between selenium and other components of MetS beyond hyperglycemia has been less studied. Consistent with our findings that higher selenium was associated with higher odds of hypertriglyceridemia and lower odds of low HDL-C, a recent study also revealed positive associations of selenium with triglyceride and HDL-C [27]. However, our findings that the inverse association between selenium and low HDL-C was significantly stronger in men than in women was contrary to a previous study that revealed that selenium was inversely related with HDL-C only in women [28]. The disagreement might be partly attributed to different study designs and population characteristics. Additionally, a few studies have suggested negative associations of selenium with waist circumference and blood pressure at low selenium status [29, 30], which were in line with our findings of U-shaped associations.

Biological mechanisms of how selenium is involved in the pathogenesis of these metabolic disorders are limited. As selenium levels affect the expression and activity of selenoproteins, selenium might be involved in these metabolic disorders via selenoproteins [31]. Suboptimal selenoproteins related to insufficient levels of selenium might result in increased ROS [32]. Also, excess levels of selenium might result in increased production of ROS via the rise of inorganic selenium in plasma [33, 34]. Then, increased ROS might result in oxidative stress and insulin resistance [35], both of which are suggested to be the central mechanisms of MetS [2]. Moreover, oxidative stress also is a feature of adiposity and hypertension [36]. In animal models, high dietary selenium intake could upregulate the expression of key factors related to gluconeogenesis and lipogenesis, suppress expression of insulin signaling-related protein, and subsequently induce dyslipidemia, hyperinsulinemia, and insulin resistance [37]. And more studies are warranted to clarify the mechanisms underlying the complex and various associations between selenium and these metabolic disorders.

In the current study, we firstly examined the relation of the SELENOP rs7579 polymorphism with MetS, and found that the GA genotype of rs7579 was associated with a higher risk of MetS. As a functional polymorphism located in the 3′-untranslated region of SELENOP, the rs7579 polymorphism was suggested to affect plasma SELENOP concentrations and the proportion of SELENOP isoforms [38]. And decreased expression of SELENOP might be accompanied with increased inorganic selenium such as selenomethionine, which may induce increased oxidative stress [33, 34, 39]. Consistently, several previous studies have correlated the rs7579 polymorphism with cancer [40, 41], which is also characterized with oxidative stress [42]. And increased oxidative stress might ultimately lead to MetS [4]. In addition, the administration of purified SELENOP was suggested to impair insulin signaling and disturb glucose metabolism, as well as play a role in the development of atherosclerosis [14, 43, 44]. However, different from the variable associations of selenium with components of MetS, no significant association between rs7579 and a component of MetS was observed in our study. As both plasma levels of selenium and the rs7579 polymorphism take part in regulating the gene expression of SELENOP [15], selenium might be involved in the development of MetS partly through SELENOP, while the relation between selenium and components of MetS might be attributed to the effect of selenium independent of SELENOP.

The strengths of the current study included the large sample size, a matched case-control study design to minimize the influence of key confounding factors, and the use of a cubic spline regression analysis rather than a simple linear analysis which enabled us to explore the nonlinear relation between selenium and MetS. Moreover, to the best of our knowledge, this was the first population-based study systematically investigating the relation of rs7579 with MetS in a large sample size, and we identified the A risk allele with MetS.

Despite these strengths, several limitations should also be considered. Firstly, the case-control study design did not allow us to establish any causality relationship. Moreover, because our participants with MetS were not confined to the newly diagnosed and drug naive, we cannot preclude the effect of drug interventions on selenium metabolism. Therefore, our findings should be interpreted with caution. Secondly, considering that our study was conducted in a selenium-appropriate area of China, most participants included were with appropriate selenium status, which precluded us to explore the associations of selenium deficiency and selenium excess with metabolic disorders. Hence, our findings may not reflect whole dose-response associations between selenium and these metabolic disorders, and the conclusions may not be generalized to population living in selenium-deficient or selenium-excessive areas. Further studies in more diverse groups with various selenium status are needed. Thirdly, dietary intake data were not available in our study, which prohibited us from conducting analyses between dietary factors and plasma selenium. However, circulating selenium is an accepted biomarker of selenium intake and is able to objectively assess dietary consumption without the bias of self-reported dietary intake errors [22]. Lastly, even if we have controlled for various confounding factors like sex, age, BMI, lifestyle factors, and education level, there might be other residual confounding factors that we did not measure but may impact the association examined, such as supplement use or socioeconomic factors.

## 5. Conclusions

Our case-control study suggested a U-shaped association between plasma selenium and MetS. Furthermore, we observed U-shaped associations of plasma selenium with central obesity and high blood pressure, positive associations of selenium with hypertriglyceridemia and hyperglycemia, and a negative association between selenium and low HDL-C. In addition, we found that the SELENOP rs7579 polymorphism was associated with MetS. Our findings may contribute to the understanding of the etiologic role of selenium in MetS development and imply the potential adverse effects of both high selenium and low selenium status. Future studies are warranted to confirm our findings in prospective studies and to elucidate the underlying mechanisms.

## Figures and Tables

**Table 1:** Characteristics of study participants^a^.

Characteristics	Non-MetS (*n* = 1279)	MetS (*n* = 1279)	*P* value
Male, *n* (%)	818 (64.0)	818 (64.0)	1.000
Age (years)	55.36 (10.53)	55.56 (10.46)	0.637
BMI (kg/m^2^)	22.78 (2.67)	25.92 (2.93)	<0.001
SBP (mmHg)	131.91 (18.51)	146.01 (21.17)	<0.001
DBP (mmHg)	77.61 (10.71)	83.64 (12.08)	<0.001
Waist circumference (cm)	80.89 (7.79)	90.14 (7.85)	<0.001
Triglycerides (mmol/L)	1.14 (0.84-1.48)	1.94 (1.50-2.62)	<0.001
Total cholesterol (mmol/L)	4.58 (4.06-5.20)	4.97 (4.37-5.68)	<0.001
HDL-C (mmol/L)	1.41 (1.21-1.62)	1.20 (0.98-1.37)	<0.001
LDL-C (mmol/L)	2.55 (2.00-3.09)	2.71 (2.04-3.34)	<0.001
Fasting plasma glucose (mmol/L)	5.63 (0.96)	6.31 (1.61)	<0.001
Current smoker, *n* (%)	433 (33.9)	396 (31.0)	0.118
Current drinker, *n* (%)	397 (31.0)	384 (30.0)	0.577
Vigorous activity (at least once/week), *n* (%)	549 (42.9)	521 (40.7)	0.262
Educational level, *n* (%)			0.175
None or elementary school	318 (24.9)	298 (23.3)	
Middle school	553 (43.2)	600 (46.9)	
High school or college	408 (31.9)	381 (29.8)	
Selenium (*μ*g/L)	92.66 (82.36-103.53)	93.88 (83.17-107.41)	0.031

^a^Data are presented as *n* (%) for categorical data, means (standard deviations) for parametrically distributed data, or medians (interquartile ranges) for nonparametrically distributed data. BMI: body mass index; DBP: diastolic blood pressure; HDL-C: high-density lipoprotein cholesterol; LDL-C: low-density lipoprotein cholesterol; MetS: metabolic syndrome; SBP: systolic blood pressure.

**Table 2:** Associations of plasma selenium concentration with MetS and its components^a^.

	Quartiles of plasma selenium concentrations (*μ*g/L)				*P* value for trend^b^	*P* value
	Q1, <82.36	Q2, 82.37-92.66	Q3, 92.67-103.52	Q4, ≥103.53		
*MetS*						
No. of cases/controls, *n*/*n*	304/320	301/320	278/319	396/320		
Crude OR	0.76 (0.61-0.95)	0.76 (0.61-0.94)	0.70 (0.56-0.87)	1	0.027	0.007
Model 1	0.80 (0.60-1.07)	0.76 (0.57-1.03)	0.62 (0.46-0.83)	1	0.266	0.017
Model 2	0.79 (0.59-1.06)	0.75 (0.56-1.01)	0.61 (0.45-0.83)	1	0.218	0.015
*Central obesity*						
No. of cases/controls, *n*/*n*	334/283	316/299	282/306	388/319		
Crude OR	0.97 (0.78-1.21)	0.87 (0.70-1.08)	0.76 (0.61-0.94)	1	0.985	0.062
Model 1	0.98 (0.73-1.30)	0.90 (0.67-1.19)	0.67 (0.50-0.90)	1	0.751	0.032
Model 2	0.98 (0.73-1.31)	0.89 (0.67-1.19)	0.67 (0.50-0.89)	1	0.755	0.031
*Hypertriglyceridemia*						
No. of cases/controls, *n*/*n*	235/387	235/385	224/368	333/382		
Crude OR	0.70 (0.56-0.87)	0.70 (0.56-0.87)	0.70 (0.56-0.87)	1	0.001	0.001
Model 1	0.74 (0.59-0.93)	0.75 (0.60-0.94)	0.71 (0.57-0.90)	1	0.012	0.010
Model 2	0.73 (0.58-0.92)	0.74 (0.59-0.93)	0.72 (0.57-0.91)	1	0.008	0.009
*Low HDL-C*						
No. of cases/controls, *n*/*n*	236/386	180/440	156/438	194/521		
Crude OR	1.64 (1.30-2.07)	1.10 (0.87-1.40)	0.96 (0.75-1.22)	1	<0.001	<0.001
Model 1	1.47 (1.15-1.88)	1.06 (0.82-1.37)	0.89 (0.69-1.16)	1	0.001	0.001
Model 2	1.42 (1.11-1.82)	1.05 (0.81-1.35)	0.88 (0.68-1.15)	1	0.003	0.003
*High blood pressure*						
No. of cases/controls, *n*/*n*	326/219	322/210	300/233	383/252		
Crude OR	0.98 (0.78-1.24)	1.01 (0.80-1.28)	0.85 (0.67-1.07)	1	0.826	0.446
Model 1	1.05 (0.82-1.36)	1.00 (0.77-1.29)	0.83 (0.65-1.07)	1	0.480	0.315
Model 2	1.08 (0.84-1.40)	1.01 (0.78-1.31)	0.82 (0.63-1.05)	1	0.335	0.181
*Hyperglycemia*						
No. of cases/controls, *n*/*n*	324/298	379/240	386/209	487/227		
Crude OR	0.51 (0.41-0.63)	0.74 (0.59-0.92)	0.86 (0.68-1.08)	1	<0.001	<0.001
Model 1	0.57 (0.45-0.72)	0.82 (0.65-1.03)	0.92 (0.73-1.17)	1	<0.001	<0.001
Model 2	0.58 (0.46-0.73)	0.82 (0.65-1.04)	0.92 (0.72-1.17)	1	<0.001	<0.001

^a^Odds ratios (95% confidence intervals) for MetS were estimated by conditional logistic regression, and ORs (95% CIs) for central obesity, hypertriglyceridemia, low HDL-C, hypertension, and hyperglycemia were estimated by binary logistic regression. Model 1 was adjusted for sex, age (years), and body mass index (kg/m^2^). Model 2 was adjusted for model 1 plus smoking (current, former, and never), drinking (current, former, and never), vigorous activity (yes or no), and education level (none or elementary school, middle school, and high school or college). HDL-C: high-density lipoprotein cholesterol; MetS: metabolic syndrome; OR: odds ratio. ^b^Tests for linear trend were conducted by using the median value for each quartile and treating it as a continuous variable.

**Table 3:** Associations of SELENOP rs7579 polymorphism with MetS and its components^a^.

	Controls, *n* (%)	Cases, *n* (%)	Crude OR (95% CI)	*P* value	Adjusted OR (95% CI)^b^	*P* value
MetS						
GG genotype	373 (57.9)	299 (54.3)	1		1	
GA genotype	216 (33.5)	208 (37.7)	1.20 (0.94-1.53)	0.140	1.42 (1.06-1.91)	0.018
AA genotype	55 (8.5)	44 (8.0)	1.00 (0.65-1.53)	0.993	1.09 (0.67-1.78)	0.725
GA+AA genotype	271 (42.1)	252 (45.7)	1.16 (0.92-1.46)	0.204	1.35 (1.03-1.77)	0.033
Central obesity						
GG genotype	320 (56.8)	342 (56.0)	1		1	
GA genotype	196 (34.8)	219 (35.8)	1.05 (0.82-1.34)	0.723	1.37 (0.97-1.92)	0.072
AA genotype	47 (8.3)	50 (8.2)	1.00 (0.65-1.53)	0.983	1.06 (0.61-1.85)	0.843
GA+AA genotype	243 (43.2)	269 (44.0)	1.04 (0.82-1.31)	0.765	1.30 (0.94-1.78)	0.109
Hypertriglyceridemia						
GG genotype	445 (57.8)	226 (53.8)	1		1	
GA genotype	265 (34.4)	155 (36.9)	1.15 (0.89-1.49)	0.277	1.14 (0.86-1.50)	0.359
AA genotype	60 (7.8)	39 (9.3)	1.28 (0.83-1.98)	0.265	1.35 (0.85-2.13)	0.207
GA+AA genotype	325 (42.2)	194 (46.2)	1.18 (0.93-1.49)	0.186	1.18 (0.91-1.52)	0.218
Low HDL-C						
GG genotype	476 (56.8)	194 (55.0)	1		1	
GA genotype	294 (35.1)	128 (36.3)	1.07 (0.82-1.39)	0.627	1.16 (0.86-1.56)	0.331
AA genotype	68 (8.1)	31 (8.8)	1.12 (0.71-1.77)	0.630	1.25 (0.76-2.06)	0.376
GA+AA genotype	362 (43.2)	159 (45.0)	1.08 (0.84-1.38)	0.558	1.18 (0.89-1.55)	0.253
High blood pressure						
GG genotype	285 (56.0)	284 (56.3)	1		1	
GA genotype	188 (36.9)	177 (35.1)	0.95 (0.73-1.23)	0.672	1.01 (0.76-1.34)	0.938
AA genotype	36 (7.1)	43 (8.5)	1.20 (0.75-1.92)	0.452	1.37 (0.83-2.27)	0.214
GA+AA genotype	224 (44.0)	220 (43.7)	0.99 (0.77-1.26)	0.909	1.07 (0.82-1.40)	0.622
Hyperglycemia						
GG genotype	227 (57.9)	443 (55.5)	1		1	
GA genotype	126 (32.1)	296 (37.1)	1.20 (0.93-1.57)	0.167	1.23 (0.93-1.62)	0.144
AA genotype	39 (9.9)	59 (7.4)	0.78 (0.50-1.20)	0.251	0.76 (0.48-1.20)	0.241
GA+AA genotype	165 (42.1)	355 (45.5)	1.10 (0.86-1.41)	0.434	1.12 (0.86-1.44)	0.403

^a^Odds ratios (95% confidence intervals) for MetS, central obesity, hypertriglyceridemia, low HDL-C, hypertension, and hyperglycemia were estimated by binary logistic regression. HDL-C: high-density lipoprotein cholesterol; MetS: metabolic syndrome; SELENOP: selenoprotein P. ^b^Adjusted for sex, age (years), body mass index (kg/m^2^), smoking (current, former, and never), drinking (current, former, and never), vigorous activity (yes or no), and education level (none or elementary school, middle school, and high school or college).

**Table 4:** Associations of plasma selenium concentrations with MetS and its components according to rs7579 genotypes^a^.

	Quartiles of plasma selenium concentrations (*μ*g/L)				*P* value	*P* value for interaction^b^
	Q1, <82.36	Q2, 82.37-92.66	Q3, 92.67-103.52	Q4, ≥103.53		
MetS						0.402
GG	0.66 (0.39-1.10)	0.83 (0.51-1.36)	0.71 (0.43-1.18)	1	0.371	
GA	0.53 (0.27-1.04)	0.65 (0.33-1.29)	0.44 (0.22-0.86)	1	0.095	
AA	0.36 (0.08-1.57)	0.67 (0.16-2.86)	1.28 (0.30-5.44)	1	0.396	
Central obesity						0.710
GG	0.77 (0.42-1.44)	0.81 (0.45-1.46)	0.77 (0.42-1.41)	1	0.796	
GA	1.02 (0.47-2.21)	0.57 (0.26-1.25)	0.61 (0.28-1.33)	1	0.297	
AA	0.45 (0.10-2.03)	2.59 (0.55-12.24)	0.87 (0.20-3.72)	1	0.282	
Hypertriglyceridemia						0.266
GG	0.60 (0.37-0.97)	0.66 (0.42-1.04)	0.53 (0.33-0.84)	1	0.035	
GA	0.47 (0.25-0.89)	0.48 (0.26-0.90)	0.56 (0.31-1.02)	1	0.051	
AA	0.31 (0.09-1.05)	0.45 (0.12-1.65)	0.60 (0.17-2.12)	1	0.272	
Low HDL-C						0.166
GG	0.89 (0.52-1.52)	0.90 (0.53-1.54)	0.78 (0.45-1.34)	1	0.841	
GA	1.71 (0.91-3.19)	1.25 (0.66-2.36)	0.86 (0.45-1.66)	1	0.178	
AA	2.15 (0.60-7.74)	0.96 (0.21-4.28)	1.60 (0.40-6.41)	1	0.610	
High blood pressure						0.876
GG	1.11 (0.67-1.85)	0.89 (0.54-1.45)	0.94 (0.57-1.55)	1	0.865	
GA	0.76 (0.41-1.43)	0.66 (0.35-1.24)	0.69 (0.38-1.25)	1	0.530	
AA	1.25 (0.29-5.43)	1.22 (0.23-6.52)	0.69 (0.14-3.49)	1	0.893	
Hyperglycemia						0.007
GG	0.89 (0.56-1.41)	1.07 (0.68-1.69)	1.44 (0.89-2.34)	1	0.284	
GA	0.57 (0.31-1.07)	0.74 (0.40-1.40)	1.11 (0.58-2.13)	1	0.165	
AA	0.14 (0.04-0.50)	0.36 (0.09-1.48)	0.52 (0.13-1.98)	1	0.023	

^a^Odds ratios (95% confidence intervals) for MetS, central obesity, hypertriglyceridemia, low HDL-C, hypertension, and hyperglycemia were estimated by binary logistic regression after adjustment for sex, age (years), body mass index (kg/m^2^), smoking (current, former, and never), drinking (current, former, and never), vigorous activity (at least once/week or no), and education level (none or elementary school, middle school, and high school or college). HDL-C: high-density lipoprotein cholesterol; MetS: metabolic syndrome. ^b^Interaction tests with multiplicative terms were performed to determine whether risks differed between the subgroups.

## Data Availability

The data used to support the findings of this study are available from the corresponding author upon request.

## Abbreviations

BMI: Body mass index
CI: Confidence interval
HDL: High-density lipoprotein cholesterol
LDL-C: Low-density lipoprotein cholesterol
MetS: Metabolic syndrome
OR: Odds ratio
PCR: Polymerase chain reaction
ROS: Reactive oxygen species
SELENOP: Selenoprotein P
SNP: Single nucleotide polymorphism.

## Supplementary Materials

Supplementary Materials Table S1: subgroup analyses of plasma selenium concentrations with risk of MetS and its components stratified by sex. Table S2: characteristics of MetS cases and controls that are genotypes of the rs7579 polymorphism. Table S3: subgroup analyses of the rs7579 polymorphism with risk of MetS and its components stratified by sex. Figure S1: the flow chart of participant recruitment and case-control selection.
