# Supplementary material for: Diverse Associations of Plasma Selenium Concentrations and SELENOP Gene Polymorphism with Metabolic Syndrome and Its Components
Source: Oxid Med Cell Longev. 2020 Apr 22;2020:5343014. doi: 10.1155/2020/5343014 (PMC7195628; doi:10.1155/2020/5343014)
Supplement: Supplementary Materials — Table S1: subgroup analyses of plasma selenium concentrations with risk of MetS and its components stratified by sex. Table S2: characteristics of MetS cases and controls that are genotypes of the rs7579 polymorphism. Table S3: subgroup analyses of the rs7579 polymorphism with risk of MetS and its components stratified by sex. Figure S1: the flow chart of participant recruitment and case-control selection. [file 5343014.f1.pdf]

Supplemental Material

Diverse associations of plasma selenium concentrations, SELENOP gene polymorphism with metabolic syndrome and its components

Li Zhou [1, 2], Cheng Luo [1, 2], Jiawei Yin [1, 2], Yalun Zhu [1, 2], Peiyun Li [1, 2], Sijing Chen [1, 2], Taoping Sun [1, 2], Manling Xie [3], Zhilei Shan [1, 2, 4], Benfeng Cao [1, 2], Xueting Hu [1, 2], Ying Rong [5], Wei Yang [1, 2], Xiaoqin Li [1, 2], Aijun Tan [6, *] and Liegang Liu [1, 2, *]

Supplementary Table S1-S3

Table of Contents

Table S1 Subgroup analyses of plasma selenium concentrations with risk of MetS and its components stratified by sex.

Table S2 Characteristics of MetS cases and controls genotyped of the rs7579 polymorphism.

Table S3 Subgroup analyses of rs7579 polymorphism with risk of MetS and its components stratified by sex.

Table S1 Subgroup analyses of plasma selenium concentrations with risk of MetS and its components stratified by sex [a]

Group (n)	Quartiles of plasma selenium concentrations (μg/L)				*P* value for interaction [b]
	Q1, <82.36	Q2, 82.37-92.60	Q3, 92.67-103.5	Q4, ≥103.53	
MetS					0.158
Male (1636)	0.83 (0.60-1.15)	0.76 (0.56-1.05)	0.66 (0.48-0.91)	1	
Female (922)	0.73 (0.49-1.10)	0.85 (0.56-1.29)	0.70 (0.45-1.07)	1	
Central obesity					0.404
Male (1610)	1.11 (0.77-1.61)	1.06 (0.74-1.52)	0.77 (0.54-1.10)	1	
Female (917)	0.79 (0.48-1.29)	0.68 (0.41-1.13)	0.50 (0.30-0.86)	1	
Hypertriglyceridemia					0.709
Male (1631)	0.75 (0.56-1.01)	0.68 (0.51-0.91)	0.65 (0.49-0.86)	1	
Female (918)	0.81 (0.55-1.20)	0.93 (0.62-1.38)	0.92 (0.61-1.39)	1	
Low HDL-C					0.003
Male (1632)	1.87 (1.34-2.62)	1.21 (0.85-1.71)	0.96 (0.67-1.37)	1	
Female (919)	0.85 (0.58-1.25)	0.74 (0.50-1.11)	0.73 (0.48-1.10)	1	
High blood pressure					0.308
Male (1448)	1.08 (0.78-1.51)	0.91 (0.66-1.25)	0.89 (0.65-1.22)	1	
Female (797)	1.24 (0.80-1.90)	1.33 (0.85-2.07)	0.69 (0.44-1.09)	1	
Hyperglycemia					0.525
Male (1633)	0.53 (0.40-0.72)	0.80 (0.59-1.08)	0.91 (0.67-1.24)	1	
Female (917)	0.62 (0.43-0.91)	0.84 (0.57-1.24)	0.97 (0.65-1.43)	1	

[a] Odds ratios (95% confidence intervals) were estimated by binary logistic regression after adjustment for sex, age (years), body mass index (kg/m^2), smoking (current, former, and never), drinking (current, former, and never), vigorous activity (at least once/week or no) and education level (none or elementary school, middle school, and high school or college). HDL-C, high-density lipoprotein cholesterol; MetS, metabolic syndrome.

[b] Interaction tests with multiplicative terms were performed to determine whether risks differed between the subgroups.

Table S2 Characteristics of MetS cases and controls genotyped of the rs7579 polymorphism [a]

Characteristics	Non-MetS (n=644)	MetS (n=551)	*P* value
Male, n (%)	466 (72.4)	384 (69.7)	0.310
Age (years)	51.20 (11.08)	49.98 (10.67)	0.053
BMI (kg/m^2)	22.71 (2.57)	25.98 (2.93)	<0.001
SBP (mmHg)	129.41 (16.57)	138.63 (19.08)	<0.001
DBP (mmHg)	78.92 (10.25)	85.85 (11.66)	<0.001
Waist circumference (cm)	81.89 (7.32)	91.35 (7.68)	<0.001
Triglycerides (mmol/L)	1.09 (0.77-1.42)	1.90 (1.44-2.61)	<0.001
Total cholesterol (mmol/L)	4.42 (3.91-5.00)	4.90 (4.25-5.57)	<0.001
HDL-C (mmol/L)	1.35 (1.18-1.53)	1.23 (0.97-1.38)	0.948
LDL-C (mmol/L)	2.45 (1.79-2.99)	2.42 (1.75-3.10)	<0.001
Fasting plasma glucose (mmol/L)	5.68 (0.92)	6.35 (1.48)	<0.001
Fasting plasma insulin (mU/L)	6.63 (4.78-9.45)	10.57 (7.60-14.66)	<0.001
HOMA-IR	1.64 (1.11-2.47)	2.87 (2.01-4.04)	<0.001
HOMA-β	62.44 (46.81-91.77)	82.89 (57.15-122.00)	<0.001
Current smoker, n (%)	264 (41.0)	210 (38.1)	0.310
Current drinker, n (%)	226 (35.1)	186 (33.8)	0.628
Vigorous activity (at least once/week), n (%)	237 (36.8)	176 (31.9)	0.078
Educational level, n (%)			0.617
None or elementary school	133 (20.7)	103 (18.7)	
Middle school	291 (45.2)	248 (45.0)	
High school or college	220 (34.2)	200 (36.3)	
Selenium (μg/L)	92.46 (82.18-104.31)	95.72 (85.55-108.89)	0.001
SELENOP rs7579			0.416
Allele G	962 (74.7)	806 (73.1)	
Allele A	326 (25.3)	296 (26.9)	

[a] Data are presented as n (%) for categorical data, means (standard deviations) for parametrically distributed data, or medians (interquartile ranges)

for nonparametrically distributed data. BMI, body mass index; DBP, diastolic blood pressure; HDL-C, high-density lipoprotein cholesterol; LDL-C, low-density lipoprotein cholesterol; MetS, metabolic syndrome; SBP, systolic blood pressure.

Table S3 Subgroup analyses of rs7579 polymorphism with risk of MetS and its components stratified by sex [a]

Group (n)	rs7579 genotypes				*P* value for interaction [b]
	GG genotype	GA genotype	AA genotype	GA+AA genotype	
MetS					0.091
Male (850)	1	1.75 (1.23-2.50)	1.25 (0.69-2.28)	1.64 (1.18-2.29)	
Female (345)	1	0.85 (0.50-1.45)	0.80 (0.33-1.95)	0.84 (0.51-1.38)	
Central obesity					0.406
Male (831)	1	1.52 (1.01-2.29)	1.12 (0.56-2.25)	1.44 (0.98-2.11)	
Female (343)	1	1.04 (0.56-1.92)	0.87 (0.33-2.31)	1.00 (0.56-1.77)	
Hypertriglyceridemia					0.656
Male (847)	1	1.16 (0.84-1.60)	1.22 (0.71-2.10)	1.17 (0.86-1.58)	
Female (343)	1	0.99 (0.56-1.76)	1.71 (0.69-4.25)	1.12 (0.66-1.89)	
Low HDL-C					0.168
Male (847)	1	1.42 (0.98-2.06)	1.40 (0.76-2.61)	1.42 (1.00-2.01)	
Female (344)	1	0.86 (0.52-1.44)	1.23 (0.52-2.93)	0.93 (0.57-1.50)	
High blood pressure					0.412
Male (735)	1	0.86 (0.62-1.19)	1.47 (0.81-2.66)	0.94 (0.69-1.29)	
Female (278)	1	1.65 (0.93-2.94)	1.06 (0.38-3.00)	1.53 (0.89-2.64)	
Hyperglycemia					0.533
Male (847)	1	1.21 (0.87-1.70)	0.87 (0.50-1.52)	1.14 (0.83-1.56)	
Female (343)	1	1.20 (0.74-1.94)	0.51 (0.22-1.20)	1.02 (0.65-1.59)	

[a] Odds ratios (95% confidence intervals) were estimated by binary logistic regression after adjustment for sex, age (years), body mass index (kg/m^2), smoking (current, former, and never), drinking (current, former, and never), vigorous activity (at least once/week or no) and education level (none or elementary school, middle school, and high school or college). HDL-C, high-density lipoprotein cholesterol; MetS, metabolic syndrome.

[b] Interaction tests with multiplicative terms were performed to determine whether risks differed between the subgroups.

Supplementary Figure S1

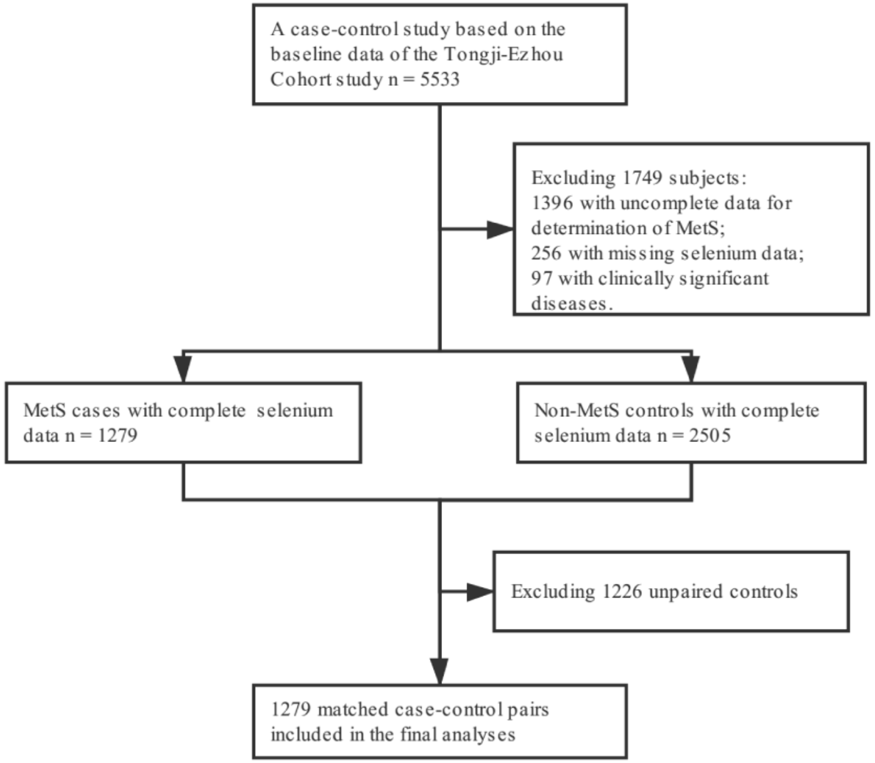

Figure S1. The flow chart of participants recruitment and case-control selection.
